# *Neorickettsia risticii *surface-exposed proteins: proteomics identification, recognition by naturally-infected horses, and strain variations

**DOI:** 10.1186/1297-9716-42-71

**Published:** 2011-06-02

**Authors:** Kathryn E Gibson, Gabrielle Pastenkos, Susanne Moesta, Yasuko Rikihisa

**Affiliations:** 1 Department of Veterinary Biosciences, The Ohio State University College of Veterinary Medicine, 1925 Coffey Rd, Columbus, OH 43210, USA

## Abstract

*Neorickettsia risticii *is the Gram-negative, obligate, and intracellular bacterial pathogen responsible for Potomac horse fever (PHF): an important acute systemic disease of horses. *N. risticii *surface proteins, critical for immune recognition, have not been thoroughly characterized. In this paper, we identified the 51-kDa antigen (P51) as a major surface-exposed outer membrane protein of older and contemporary strains of *N. risticii *through mass spectrometry of streptavidin-purified biotinylated surface-labeled proteins. Western blot analysis of sera from naturally-infected horses demonstrated universal and strong recognition of recombinant P51 over other *Neorickettsia *recombinant proteins. Comparisons of amino acid sequences for predicted secondary structures of P51, as well as *Neorickettsia *surface proteins 2 (Nsp2) and 3 (Nsp3) among *N. risticii *strains from horses with PHF during a 26-year period throughout the United States revealed that the majority of variations among strains were concentrated in regions predicted to be external loops of their β-barrel structures. Large insertions or deletions occurred within a tandem-repeat region in Ssa3. These data demonstrate patterns of geographical association for P51 and temporal associations for Nsp2, Nsp3, and Ssa3, indicating evolutionary trends for these *Neorickettsia *surface antigen genes. This study showed *N. risticii *surface protein population dynamics, providing groundwork for designing immunodiagnostic targets for PHF.

## Introduction

Discovered in 1984, *Neorickettsia *(formerly *Ehrlichia*) *risticii *is an obligate intracellular bacterium and the causative agent of Potomac horse fever (PHF) [1-3]. The bacterium uses a digenetic trematode to survive and proliferate in its natural lifecycle [4-9]. It is through accidental ingestion of the metacercarial stage of the digenetic trematode within its insect host that the horse becomes infected with *N. risticii *and develops PHF [6]. PHF is an acute, severe, and potentially fatal disease of horses, normally contracted during the summer months in North America when aquatic insect larvae infested with *N. risticii*-infected digenetic trematodes molt and emerge (hatch) from the water as adults [6,10]. Clinical signs range from mild (anorexia, fever, lethargy, and depression) to life-threatening (laminitis, abortion, and diarrhea followed by severe dehydration) [10,11]. The administration of tetracyclines at the early stage of infection is effective, in part by inhibiting bacterial protein synthesis and facilitating lysosome fusion with inclusions containing *N. risticii *[12-15]. Diagnosis of this disease is mainly done by indirect fluorescent-antibody (IFA) test based on *N. risticii*-infected cells and by nested polymerase chain reaction (PCR) on blood samples [5,16-22]. The only available vaccines are bacterins using the 1984 *N. risticii *type strain, which demonstrate inadequate efficacy [23,24].

It was determined that *N. risticii *has similar genetic, antigenic, and morphologic characteristics to *Neorickettsia helminthoeca *[25,26], which were the major reasons it, as well as *Neorickettsia *(formerly *Rickettsia*, *Ehrlichia*) *sennetsu*, was regrouped into the genus *Neorickettsia *[27]. In addition, the bacterial parasite, known as the *Stellantchasmus falcatus *(SF) agent, isolated from metacercariae in fish from Japan and Oregon [28-30] belongs to this group. *N. risticii *also consists of a variety of strains, based on PCR and sequencing of 16S RNA and *groEL*, Western blot analyses using purified bacteria as antigen, and morphology [20,22,24,31].

Little is known about *N. risticii *surface-exposed proteins, and this missing information is crucial in the understanding of bacterium-host cell interactions. Antigenic and potential surface proteins ranging between 28 and 110-kDa in mass were previously detected by Western blotting, but these proteins were not identified [32]. Immunoprecipitation of *N. risticii *labeled with I^125 ^and *N. risticii *immune mouse sera revealed potential surface proteins ranging from 25 to 62-kDa in mass, although these proteins were not identified [33]. Antigenic proteins of 70, 55, 51, and 44-kDa masses have been demonstrated utilizing recombinant proteins; again the proteins were not identified [34]. Two highly-immunodominant proteins in two *N. risticii *strains were identified as GroEL and the 51-kDa antigen (P51) [35], but it was not shown whether these proteins were surface exposed. Strain-specific antigen (Ssa) was suggested as a surface immunogenic protein with potential use in vaccine production, although it was not determined to be bacterial surface exposed [24,36].

The identification of *Neorickettsia *proteins is now achievable with the availability of whole genome sequencing data on both the type strain (Miyayama) of *N. sennetsu *[37] and the type strain (Illinois) of *N. risticii *[38]. In this paper, we determined 1) major surface proteins by proteomics analysis on *N. risticii*, 2) horse immune recognition of *N. risticii *surface proteins, and 3) strain variations in aligned sequences of these major surface proteins with respect to their predicted secondary structures.

## Materials and methods

### Culturing and isolation of *N. risticii *strains

*N. risticii *Illinois^T ^[3] and a Pennsylvania strain (PA-1) [6] were cultured in P388D_1 _cells in 75-cm^2 ^flasks containing RPMI 1640 (Mediatech, Inc., Herdon, VA, USA) supplemented with 5-10% fetal bovine serum (FBS) (U.S. Biotechnologies, Inc., Pottstown, PA, USA) and 4-6 mM L-glutamine (Invitrogen, Carlsbad, CA, USA) at 37°C under 5% CO_2_. *N. risticii *was isolated from highly-infected P388D_1 _cells as previously described for *N. sennetsu *Miyayama^T ^[39].

### Biotinylation and streptavidin-affinity purification of *N. risticii *surface proteins

Biotinylation of purified *N. risticii *Illinois and PA-1 from twenty-five 75-cm^2 ^flasks using EZ Link Sulfo-NHS-SS-Biotin (Pierce Biotechnology, Rockford, IL, USA) and subsequent bacterial lysis and collection of solubilized bacterial proteins were performed as previously described [39]. Streptavidin purification of Sulfo-NHS-SS-Biotinylated *N. risticii *proteins was then performed, followed by SDS-polyacrylamide gel electrophoresis (PAGE) and fixation and GelCode blue (Pierce) staining of the gel [39]. Proteins from seven bands from *N. risticii *Illinois and proteins from four bands or band collections from PA-1 were identified by capillary-liquid chromatography-nanospray tandem mass spectrometry (Nano-LC/MS/MS) as previously described [40].

### Western blotting using recombinant proteins

Recombinant P51 (rP51, 57 kDa), cloned from *N. risticii *Illinois (NRI_0235), and rNsp2 (35 kDa) and rNsp3 (28 kDa), cloned from *N. sennetsu *Miyayama (NSE_0873 and NSE_0875, respectively), were expressed by transformed BL21(DE3) cells using isopropyl-β-D-thiogalactopyranoside induction and His-tag purified as described previously [30,39]. Recombinant GroEL (55 kDa), derived from *N. sennetsu *Miyayama (NSE_0642), was acquired from stored aliquots [41]. Fifty μg of each recombinant protein were separated by SDS-PAGE, transferred to nitrocellulose membranes, and cut into strips. Western blotting was then performed on these strips using 1:500 dilutions of known positive horse sera samples as determined by IFA [16,21]. The membrane was subsequently incubated with a 1:1000 dilution of horseradish peroxidase-conjugated goat anti-horse (Kirkegaard & Perry Laboratories, Inc., Gaithersburg, MD, USA) as secondary antibody. Enhanced chemiluminescence (ECL) LumiGLO chemiluminescent reagent (Pierce) and a LAS3000 image documentation system (FUJIFILM Medical Systems USA, Stamford, CT, USA) were used to visualize the protein bands with 300 s exposure. Bands were aligned using Precision Plus prestained protein standards (Bio-Rad Laboratories, Hercules, CA, USA).

### Polymerase chain reaction, sequencing, and sequence alignment

DNA was purified from buffy coats of PHF-positive horses or cultures of *N. risticii *in P388D_1 _cells using the DNeasy Blood and Tissue Kit (QIAGEN, Valencia, CA, USA), according to manufacturer's instructions. PCR amplification was then performed using either Phusion or Taq DNA polymerase (New England BioLabs, Ipswich, MA, USA) and primers designed for conserved regions through alignment of multiple *Neorickettsia *spp. and/or *N. risticii *strains (see Additional file 1). Sequencing was performed by The Ohio State University Plant-Microbe Genomics Facility. Sequences containing whole genes or gene fragments were translated and aligned mainly through the CLUSTAL W (slow/accurate) method in the MegAlign program of DNAStar (DNAStar, Madison, WI, USA); P51 was first aligned by CLUSTAL V (PAM250) method, and Ssa3 was aligned both by CLUSTAL W and manually. External loops were also aligned separately by CLUSTAL W for both P51 and Nsp3. Amino acid (aa) variations in *N. risticii *strains and other *Neorickettsia *spp. for all proteins were determined in relation to *N. risticii *Illinois. Protein alignments of the same size (including deletions as dashes) were analyzed by PHYLIP (v3.66) to obtain bootstrap values for 1000 replicates (using the programs SeqBoot, Protdist, Neighbor, and Consense) and to create dendrograms (using the programs Protdist, Neighbor, and Drawgram) [42]. Protein properties, including antigenicity profiles and β-sheet predictions were determined using the Protean program (DNAStar). Gene and protein sequence homologies were also demonstrated using Basic Local Alignment Search Tool (BLAST) algorithms, including blastn, protein-protein blastp, and blastp [43,44].

### Prediction of secondary structures

Predictions for Nsp2 and Nsp3 were based on a combination of the programming algorithm in the PRED-TMBB web server [45], hydrophobicity and hydrophobic movement profiles [46], and DNAStar MegAlign (DNAStar, Madison, WI, USA) alignment and analyses of all available *N. risticii *strain and *Neorickettsia *spp. sequences.

### GenBank Accession Numbers

GenBank accession numbers of all sequences determined in this study are shown in Table 1. P51 sequences previously deposited in GenBank used in this study are listed in Table 2. Nsp2 sequences include *N. risticii *Illinois (NRI_0839, YP_003082043) and *N. sennetsu *Miyayama (YP_746740). Previously-deposited Nsp3 sequences include *N. risticii *Illinois (NRI_0841, YP_003082045) and *N. sennetsu *Miyayama (YP_506742). Ssa3 sequences include *N. risticii *Illinois (NRI_0872, YP_003082075) and *N. sennetsu *Miyayama (NSE_0908, YP_506773). The Ssa1 sequence is from *N. risticii *Illinois (NRI_0870, YP_003082073), and other Ssas are from 25-D (AAC31427) and 90-12 (AAC31428).

**Table 1:** Sequences amplified for *Neorickettsia*.

**Sample ID**^**a**^	Location/Year	**Fragment size (bp)**^**b**^	**Gene(s) amplified**^**c**^	**Accession no**.
PA-1	Pennsylvania/2000	2091	*nsp2*, *nsp3*	HQ857586
		765	*ssa1 *(p)	HQ857584
		1812	*ssa3*	HQ857585

Herodia	Pennsylvania/1999	673	*p51 *(p)	HQ857589
		2133	*nsp2*, *nsp3*	HQ857588
		1460	*ssa3*	HQ857587

081	Ohio/1991	2420	*nsp2*, *nsp3*	HQ857591
		717	*ssa3 *(p)	HQ857590

MN	Minnesota/2002	676	*p51 *(p)	HQ857594
		2156	*nsp2*, *nsp3*	HQ857593
		1029	*ssa3 *(p)	HQ857592

OV	Kentucky/1993	2103	*nsp2*, *nsp3*	HQ857596
		863	*ssa3*	HQ857595

IA03-1	Iowa/2003	1550	*nsp2 *(p), *nsp3*	HQ875741

IL01-1	Illinois/2001	623	*nsp2 *(p)	HQ875742
		489	*nsp3 *(p)	HQ875743

IN01-1	Indiana/2001	1879	*nsp2 *(p), *nsp3*	HQ875744

IN02-1	Indiana/2002	2052	*nsp2 *(p), *nsp3*	HQ875745

IN02-2	Indiana/2002	542	*p51 *(p)	HQ875747
		733	*nsp3 *(p)	HQ875746

IN03-1	Indiana/2003	542	*p51 *(p)	HQ906674
		2110	*nsp2*, *nsp3*	HQ906673

IN03-2	Indiana/2003	1361	*nsp2*, *nsp3 *(p)	HQ906675

KY03-1	Kentucky/2003	673	*p51 *(p)	HQ906678
		594	*p51 *(p)	HQ906679
		306	*p51 *(p)	HQ906680
		2095	*nsp2*, *nsp3*	HQ906677
		1129	*ssa3 *(p)	HQ906676

KY03-2	Kentucky/2003	1398	*nsp2*, *nsp3 *(p)	HQ906681

KY03-3	Kentucky/2003	1042	*nsp2 *(p), *nsp3 *(p)	HQ906682

OH07-1	Ohio/2007	259	*p51 *(p)	HQ906685

		721	*ssa1 *(p)	HQ906683
		1739	*ssa3*	HQ906684

OH07-2	Ohio/2007	259	*p51 *(p)	HQ906686

OH07-3	Ohio/2007	1558	*nsp2 *(p), *nsp3 *(p)	HQ906688

		995	*ssa3 *(p)	HQ906687
OH07-4	Ohio/2007	654	*p51 *(p)	HQ906691
		1118	*nsp2 *(p), *nsp3 *(p)	HQ906690
		1029	*ssa3 *(p)	HQ906689

OH10-1	Ohio/2010	768	*ssa3 *(p)	HQ906692

OH10-2	Ohio/2010	660	*p51 *(p)	HQ906693

TN02-1	Tennessee/2002	676	*p51 *(p)	HQ906695
		622	*p51 *(p)	HQ906696
		1893	*nsp2 *(p), *nsp3*	HQ906694

SF Oregon	Oregon/2004	1171	*nsp2*	HQ906697
		842	*nsp3*	HQ906698
		370	*ssa3 *(p)	HQ906699

**^a^**All samples, except for PA-1 and SF Oregon are from naturally-infected horses. PA-1 is an isolate from an experimental equine infection utilizing *N. risticii*-infected insects from Pennsylvania [6]. Both 081 and OV are strains of *N. risticii *previously described and with unique morphologies and sequences [5,20,22]. SF Oregon is a strain of the *Stellantchasmus falcatus *agent [30].

**^b^**The largest fragment size acquired containing the given gene(s) is shown. Multiple fragments may be present for a sample.

**^c^**p, partial sequence for the given gene was obtained.

**Table 2:** GenBank P51 sequences used in this study.

Sample ID	**Accession no.**^**a**^	Sample ID	**Accession no**.
*N. risticii *Illinois^T^	YP_003081464	11908	AAL79561
PA-1	AAM18377	SF Hirose	AAL12490
PA-2	AAM18376	SF Oregon	AAR23988
Eclipse	AAC01597	Dr. Pepper	AAC01596
SqCaddis	AAM18381	Ms. Annie	AAC01599
SqMouse	AAM18380	SHSN-1	AAB95417
S21	AAG03352	SHSN-2	AAB95418
TW2-1	AAR22503	SRC	AAB95419
TW2-2	AAR22504	SCID/CB17	AAG09962
25-D	AAB46983	Snail 2121	AAF20073
90-12	AAB46982	CF1-snail 2121	AAF20072
CM1-1	AAR22501	Shasta-horse	AAF43112
081	AAG03354	Caddis-1	AAF26718
OV	AAG03353	Caddis-2	AAF26748
Doc	AAC01595	Siskiyou horse-1	AAF20069
Oregon	AAC01600	Siskiyou horse-2	AAF20070
*N. sennetsu *Miyayama^T^	YP_506136	Siskiyou horse-3	AAF20071
Kawano	AAR23991	Juga-1	AAC01598
Nakazaki	AAR23990	Stonefly-1	AAF26749

**^a^**All sequences listed are P51 sequences that have been previously deposited in GenBank. *N. sennetsu *Miyayama P51 is NSE_0242.

## Results

### Nano-LC/MS/MS of streptavidin-affinity purified surface proteins

Given that only the *N. risticii *Illinois genome (NC_013009) has been sequenced [38], these data were used for proteomic analyses. Four *N. risticii *proteins in *N. risticii *Illinois (1984 isolate) and five *N. risticii *proteins (with conserved peptide sequences in relation to *N. risticii *Illinois) in PA-1 (2000 isolate) contained two or more peptide queries identified by Nano-LC/MS/MS (Table 3). Proteins identified for *N. risticii *Illinois were P51, GroEL (NRI_0614), Nsp3, and a conserved hypothetical protein (NRI_0567). The largest protein coverage and the largest number of peptides identified were both from P51. Proteins identified in PA-1 also included P51 and GroEL; the largest number of peptides was from P51. Minor proteins identified in PA-1 strain were DnaK (NRI_0017), ATP synthase F1, alpha subunit (AtpA, NRI_0132), and strain-specific antigen 3 (Ssa3, NRI_0872).

**Table 3:** Proteomics-identified proteins for two *N.risticii *strains.

Locus ID	Protein name	Mol Mass (kDa)	**pI**^**a**^	**% (query) peptide coverage**^***b***^	**Signal peptide**^**c**^
***N. risticii *Illinois^T^**					

NRI_0235	51-kDa antigen (P51)	54.9	8.44	49.2 (139)	Yes (20-21)
NRI_0614	Heat shock protein 60 (GroEL)	58.1	5.23	43.2 (36)	No
NRI_0841	*Neorickettsia *surface protein 3 (Nsp3)	25.7	5.96	12.0 (2)	Yes (24-25)
NRI_0567	Conserved hypothetical protein	50.9	4.26	9.85 (2)	No

**PA-1**					

NRI_0235	P51	54.9	8.44	34.6 (41)	Yes (20-21)
NRI_0614	GroEL	58.1	5.23	45.6 (36)	No
NRI_0017	Heat shock protein 70 (DnaK)	68.4	5.18	2.20 (6)	No
NRI_0132	ATP synthase F1, alpha subunit (AtpA)	55.9	5.29	2.75 (3)	No
NRI_0872	Strain-specific surface antigen 3 (Ssa3)	41.9	6.01	2.36 or 4.72**^d ^**(2)	No

**^a^**Theoretical isoelectric point of the given protein as predicted by ExPASy Compute pI/MW tool [64].

**^b^**Indicates percentage coverage of proteins by all peptides. Numbers in parentheses are the number of peptide queries for each protein identified in the given band.

**^c^**Signal peptide presence as determined by the Center for Biological Sequence Analysis SignalP v.3.0 [65]. Parentheses indicate amino acids between which cleavage is predicted to occur in the given protein.

**^d^**The peptide detected twice was within the repeated region of Ssa3, therefore the percentage coverage could be two different percentages.

### Immune recognition of major surface antigens by PHF-positive horse sera

**Figure 1:**
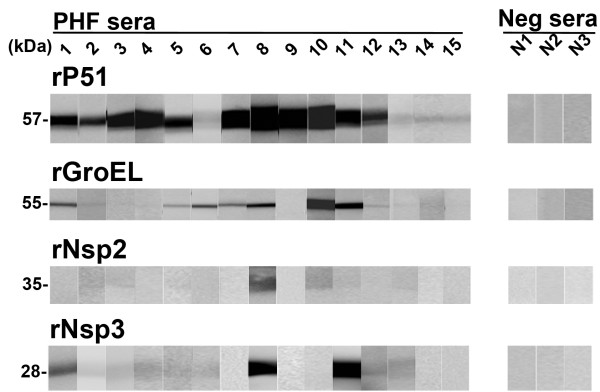
**Western blotting against rP51, rGroEL, rNsp2, and rNsp3 using PHF-positive equine sera**. Recombinant P51, rGroEL, rNsp2, and rNsp3 were separated by SDS-PAGE and probed with 1:500 dilution of PHF-positive horse sera (PHF sera, 1-15) and negative sera (Neg sera, N1-N3). Molecular masses are shown for each recombinant protein. Information regarding the sera samples is given in Table 4.

Bacterial surface-exposed proteins are generally major antigens [47]. Though only Nsp3 was detected on the surface of *N. risticii *Illinois by nano-LC/MS/MS, rNsp2 was included in the Western blotting studies because both Nsp3 and Nsp2 from *N. sennetsu *Miyayama are significant surface proteins (Figure 1, Table 4) [39]. All 15 PHF-positive samples demonstrated recognition of rP51, with 11 out of 15 sera having strong recognition. *N. sennetsu *Miyayama GroEL is 98% identical to *N. risticii *Illinois GroEL, and antisera to rGroEL of *N. sennetsu *cross-reacts with GroEL from multiple species of Rickettsiales, including *N. risticii *[41]. Six out of 15 PHF-positive serum samples demonstrated strong reactivity to rGroEL, with the rest having weak to no reactivity. Nsp2 and Nsp3 from *N. sennetsu *Miyayama are 83% and 84% identical to Nsp2 and Nsp3 from *N. risticii *Illinois, respectively, using protein-protein blastp. Only one serum sample reacted strongly to rNsp2, with the rest having weak to no reactivity. Three sera reacted strongly to rNsp3, with the rest having weak to no reactivity. All negative controls did not recognize any of the recombinant proteins.

**Table 4:** PHF-positive sera from naturally-infected horses and negative sera.

**Horse ID**^**a**^	**Clinical signs**^**b**^	Location	Year	IFA titer
**1 (OH10-1)**	A, F, De, Deh, C	Johnstown, OH	2010	> 1:10,240
**2 (OH10-2)**	A, F, De, C, L, Et, EUTH	Grove City, OH	2010	> 1:10,240
**3**	A, F, De, Deh, L, Et, EUTH	Richwood, OH	2010	> 1:10,240
**4**	A, De, F	Galloway, OH	2010	> 1:10,240
**5**	A, De, Deh, F, C, L	Dayton, OH	2010	> 1:10,240
**6**	A, F, C, L, EUTH	Loveland, OH	2010	> 1:10,240
**7**	U	Indiana	2010	1:5120
**8**	A, Di, De, Deh, F, L	Troy, OH	2008	1:1280
**9**	U	Kentucky	2008	1:1280
**10**	U	Indiana	2008	1:1280
**11**	A, F, Di, De, Deh	Columbus, OH	2008	1:1280
**12**	A, F, Di	Cattaraugus, NY	2010	1:640
**13**	U	Indiana	2008	1:640
**14**	A, F, C	Oak Hill, OH	2008	1:80
**15**	A, F	Utica, OH	2008	1:80
**N1**	U	New Jersey	2010	< 1:20
**N2**	U	Ohio	2010	< 1:20
**N3**	U	New Jersey	2010	< 1:20

**^a^**Sera 1 and 2 are from the same horses as buffy coats OH10-1 and OH10-2, respectively, as identified in Table 1.

**^b^**A, anorexia; F, fever; De, depression; Deh, dehydration; C, colic; L, laminitis; Et, endotoxemia; EUTH, euthanized; U, Unknown; Di, diarrhea.

### Sequence variation in P51

**Figure 2:**
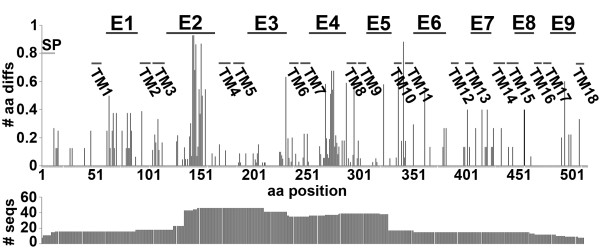
**P51 amino acid sequence variations**. Amino acids different from ***N. risticii ***Illinois, including insertions and deletions are divided by the number of sequences plotted for each amino acid position (# aa diffs). The horizontal axis displays P51 amino acid positions (aa position) including the signal peptide and all detected amino acid insertions (515 aa total). SP, signal peptide. E, external loop; and TM, transmembrane domain are based on the predicted secondary structure [39]. The number of sequences available at each amino acid position on P51 (# seqs) is shown below.

**Figure 3:**
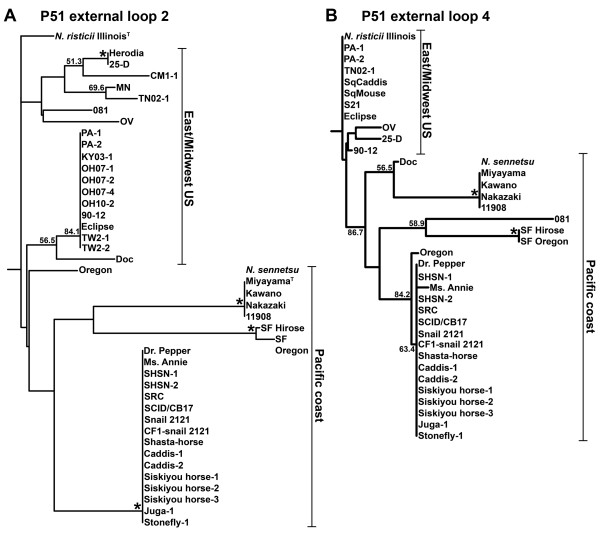
**P51 amino acid sequence variations among *Neorickettsia *sequences**. Dendrograms of P51 from (A) a 41-aa fragment (counting all insertions) including the majority of predicted external loop 2 with 43 sequences and (B) a 31-aa fragment (counting all insertions) including the entire predicted external loop 4 with 36 sequences are shown with bootstrap values greater than 50.0% for 1000 replicates. *, bootstrap value of 90.0% or greater. East/Midwest US, sequences from states in the Eastern and Midwestern US. Pacific coast, sequences from Japan, Malaysia, and US states bordering the Pacific Ocean. GenBank accession numbers for P51 sequences are listed in Tables 1 and 2.

P51 sequences are known to be strain variable [5,30]. Since P51 was found to be the major target of horse immune recognition, we examined in which part of the P51 molecule sequence variations occur. *N. sennetsu *P51 was predicted to have 18 transmembrane β-barrel proteins with nine external loops [39]. *N. sennetsu *and the SF agent, which are closely-related to *N. risticii *[28,30,48] were included for comparison. P51 alignments of a total of 52 sequences and sequence fragments from *N. risticii *during a 26-year period throughout the United States revealed high variability within regions corresponding to external loops 2 and 4 (Figure 2). Forty-three P51 sequence fragments (aa 136-176) containing most of external loop 2 (aa 120-176), and 36 P51 sequence fragments (aa 259-286) containing the entire external loop 4 were analyzed using PHYLIP (Figure 3a and 3b). Both loops 2 and 4 created patterns of clustering for sequences from states in the Eastern and Midwestern United States (East/Midwest US) and sequences from Japan, Malaysia, and US states bordering the Pacific Ocean (Pacific coast). The California strain Doc and the Ohio strain 081 did not follow this pattern, both being in East/Midwest US for external loop 2 and in Pacific coast for external loop 4. In external loop 2, *N. risticii *Illinois was only loosely associated with the other East/Midwest US sequences; in external loop 4, *N. risticii *Illinois tightly clustered with several East/Midwest US sequences. External loop 4 of 081 clustered with the SF agent strains rather than with other *N. risticii *strains.

### Sequence variation in Nsp2

**Figure 4:**
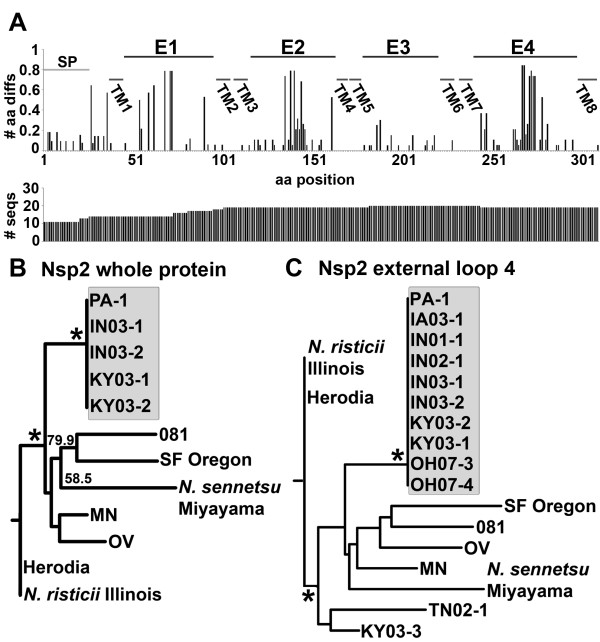
**Nsp2 amino acid sequence variations**. (A) Amino acids different from ***N. risticii ***Illinois, including insertions and deletions are divided by the number of sequences plotted for each amino acid position (# aa diffs). The horizontal axis displays Nsp2 amino acid positions (aa position) including the signal peptide and all detected amino acid insertions (309 aa total). SP, signal peptide. E, external loop; and TM, transmembrane domain are based on the predicted secondary structure. The number of sequences available at each amino acid position on Nsp2 (# seqs) is shown below. (B) Dendrograms of Nsp2 from the full-length protein, including the signal peptide (12 sequences total) and (C) the predicted external loop 4 (55 aa, including all insertions; 19 sequences total) are shown with bootstrap values greater than 50.0% for 1000 replicates. *, bootstrap value of 90.0% or greater. Post-2000 sequences are shown in the shaded area. GenBank accession numbers of new sequences are listed in Table 1.

Nsp2 sequences of *N. risticii*, other than the sequence from *N. risticii *Illinois, have not been determined. Nsp2 was predicted to have eight transmembrane β-barrel domains with four external loops. A total of 20 Nsp2 proteins and protein fragments were aligned. Amino acid variations were determined in relation to *N. risticii *Illinois. Variations mainly occurred in external loops, with the most variation occurring within external loop 4 (Figure 4a). Full-length Nsp2 (including the signal peptide), with 11 sequences total, as well as the external loop 4 region (aa 244-297) with 19 sequences total were analyzed by PHYLIP (Figure 4b and 4c). For full-length Nsp2 and external loop 4, most *N. risticii *strains obtained after the year 2000 (post-2000 strains, Table 1) were 100% identical, whereas other strains were more diverse (Figure 4b and 4c). Nsp2 for both *N. risticii *Illinois and Herodia (which were 100% identical) were unique to all other *N. risticii *strains. For full-length Nsp2, 081 clustered with SF Oregon, rather than with other *N. risticii *strains. Additionally, external loop 2 (also demonstrating high variation) showed similar patterns of clustering as seen in full-length Nsp2 and external loop 4; the exceptions were MN, which was 100% identical to *N. risticii *Illinois and Herodia, and OH07-4, which had one amino acid difference in comparison to the majority of post-2000 strains in this region (data not shown).

### Sequence variation in Nsp3

**Figure 5:**
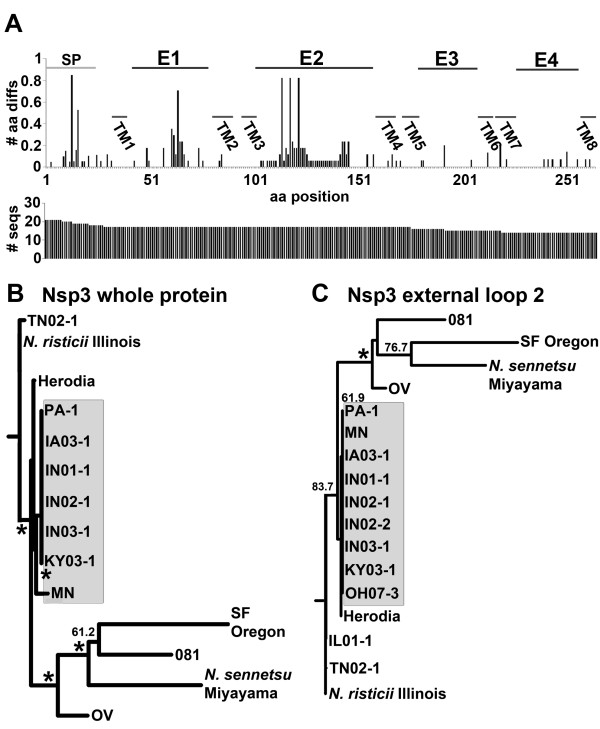
**Nsp3 amino acid sequence variations**. (A) Amino acids different from ***N. risticii ***Illinois, including insertions and deletions are divided by the number of sequences plotted for each amino acid position (# aa diffs). The horizontal axis displays Nsp3 amino acid positions (aa position) including the signal peptide and all detected amino acid insertions (264 aa total). SP, signal peptide. E, external loop; and TM, transmembrane domain are based on the predicted secondary structure. The number of sequences available at each amino acid position on Nsp3 (# seqs) is shown below. (B) Dendrograms of Nsp3 from the full-length protein, including the signal peptide (14 sequences total) and (C) the predicted external loop 2 (57 aa, including all insertions; 17 sequences total) are shown with bootstrap values greater than 50.0% for 1000 replicates. *, bootstrap value of 90.0% or greater. Post-2000 sequences are shown in the shaded area. GenBank accession numbers of new sequences are listed in Table 1.

Nsp3 sequences of *N. risticii*, except for the sequence from *N. risticii *Illinois have also not been determined. Nsp3 was predicted to have eight transmembrane β-barrel proteins with four external loops. Alignment of a total of 21 Nsp3 proteins and protein fragments demonstrated the highest variation within predicted external loop 2, yet there was less variation in the C-terminal region comprising external loops 3 and 4 (Figure 5a). Fourteen full-length Nsp3 sequences (including signal peptides) and 17 external loop 2 regions (aa 102-136) were analyzed by PHLYIP (Figure 5b and 5c). As seen in Nsp2, *N. risticii *Illinois had marked differences to other sequences, in particular to most post-2000 strains (Table 1). TN02-1 and IL01-1 had the highest similarity to *N. risticii *Illinois.

### Sequence variation in Ssa3

**Figure 6:**
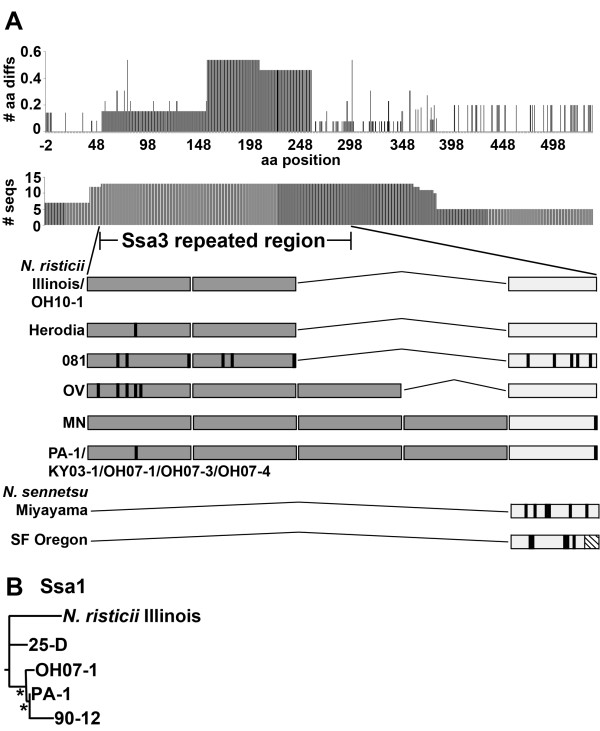
**Ssa amino acid variations and repeated regions**. (A) Ssa3 changes in amino acids, including insertions and deletions were divided by the number of sequences for each amino acid position (# aa diffs). The length of Ssa3 (horizontal axis) displays Ssa3 amino acid positions (aa position) and includes all amino acid insertions (537 aa total), and the number of sequences available at each amino acid position on Ssa3 (# seqs) is given. The amplified Ssa3 repeat region location (aa 53-196, in relation to ***N. risticii ***Illinois) and variety are demonstrated below. Dark gray boxes indicate the 52-aa repeats found in the ***N. risticii ***sequences. Light gray boxes indicate the terminal 40-aa peptide found in all ***Neorickettsia ***sequences. Black lines indicate amino acid variations in relation to ***N. risticii ***Illinois. The box containing diagonal lines in SF indicates a 9-aa truncation in the 40-aa peptide in relation to the other ***Neorickettsia ***spp. (B) The dendrogram of a 241-aa fragment of Ssa1, including all insertions (five sequences total) is shown. *, bootstrap value of 90.0% or greater. GenBank accession numbers of new sequences are listed in Table 1.

Ssa3 sequences of *N. risticii*, other than that of *N. risticii *Illinois have not been ascertained. Ssa3 was included in the analysis, since unknown Ssas were previously reported as major *N. risticii *surface antigens in the 1984 Maryland strain 25-D and the 1990 Maryland strain 90-12 [31], and a small amount Ssa3 was detected in both *N. risticii *PA-1 in this study and in *N. sennetsu *Miyayama [39]. There was no signal peptide predicted for Ssa3 [38], and Ssa3 was not predicted to have a β-barrel structure. It was originally shown that *ssa*s contain a wide variety of mainly small repeats of 10-55 bp in size [31]. Tandem repeats ranging in size from 63-156 bp are present in *ssa1*, *ssa2*, and *ssa3 *of *N. risticii *Illinois [38]. In particular, the N terminus of Ssa3 contains 2.2 copies of a 52-aa (156 bp) tandem repeat in *N. risticii *Illinois (aa 53-196) [38]. Thirteen Ssa3 proteins and protein fragments were aligned and compared (Figure 6a). Within this N-terminal repeated region, *Neorickettsia *spp. consisted of anywhere from zero to four repeated 52-aa peptides arranged in tandem followed by a terminal 40-aa peptide similar to the 52-aa repeats (for *N. risticii *Illinois: 50% identical, E-value = 6 × 10^-8^, using protein-protein blastp). It appears that the number of 52-aa repeats increases over time; six post-2000 strains (Table 1) have four repeats. There is further variety in the form of point mutations within the 52-aa repeats and terminal 40-aa peptide. In addition, the terminal 40-aa peptide in SF Oregon was truncated by 9 aa (31 aa in length, with the downstream sequence aligning with the other *Neorickettsia *sequences downstream of their terminal 40-aa peptides). Of note, there are β-sheets predicted to encompass most of the repeated region (aa 40-67; 76-119; 128-167) and scattered within the C-terminal region (aa 235-433).

### Sequence variation in Ssa1

Ssa1 sequences of *N. risticii*, other than that of *N. risticii *Illinois have not been determined. Given the strongest similarities between *ssa1 *of *N. risticii *Illinois and the unknown *ssa*s from *N. risticii *strains 25-D (isolated in 1984) and 90-12 (isolated in 1990) [38], two *ssa1 *fragments were amplified, sequenced, and translated from PA-1 and OH07-1. PA-1 (aa 11-249) and OH07-1 (aa 1-239) Ssa1 fragments were aligned with corresponding regions from *N. risticii *Illinois Ssa1 (aa 246-469) and the Ssas from 25-D (aa 287-507) and 90-12 (aa 579-817). Ssa1 fragments from PA-1 and OH07-1, which are both post-2000 strains, clustered with the 90-12 Ssa, rather than with the 1980s isolates *N. risticii *Illinois Ssa1 and 25-D Ssa, suggesting a chronological trend (Figure 6b).

## Discussion

The genes *p51, nsp2, nsp3*, and *ssa3 *are uniquely evolved in *Neorickettsia *spp. The gene *p51 *is a single copy gene and demonstrates only loose associations with other proteins of the family Anaplasmataceae [37,38]. The *nsp*s and *ssa*s are both potential operons, consisting of three genes tandemly arranged [38]. The *nsp*s belong to pfam01617, and similar to *Ehrlichia chaffeensis omp-1 *(*p28*) genes (also from pfam01617) [49], the proteins encoded by *nsp2 *and *nsp3 *were strain variable. As seen in the *ssa*s, other members of the family Anaplasmataceae have genes encoding proteins containing strain-variable tandem repeats (involving amino acid variation and changes in the numbers of tandem repeats), including Trp120 (formerly gp120), Trp47 (formerly gp47), and VLPT (variable-length PCR target) from *E. chaffeensis *and Trp140 (formerly gp140), Trp36 (formerly gp36), and gp19 from *Ehrlichia canis *[50-52]. Of note, the proteins encoded by the *ssa*s are not homologous to any proteins of the family Anaplasmataceae by blastp. Among *p51*, the *nsp*s, and the *ssa*s, there have been no signs of intragenomic recombination events, which are seen in the *Anaplasma p44/msp2 *expression locus [53,54].

Proteomics results performed on two strains of *N. risticii *established that P51 is a dominant surface-expressed protein. The recognition of recombinant P51 by PHF horse sera, even by 1:80 IFA titer sera suggests P51 is expressed and highly recognized within the present day naturally-infected horses. Despite P51 amino acid sequence variation among *N. risticii *strains, this strong universal recognition by horse immune sera suggests rP51 may serve as a defined serodiagnostic antigen. Furthermore, the study suggests that there are immunodominant conserved peptide sequences within P51 which might serve as even more specific PHF diagnostic antigens.

Sequence comparison of these surface-exposed proteins of *N. risticii *strains, with respect to the predicted protein secondary structure, the majority of which are clinical isolates, indicates there are hot spots within the genes with greater strain divergence. These include external loops 2 and 4 in P51, external loop 4 in Nsp2, external loop 2 in Nsp3, and the repeated region of Ssa3. P51 showed strong geographical association; and Nsp2, Nsp3, and Ssa3 showed temporal association. Importantly, *N. risticii *Illinois (upon which vaccines for PHF are produced) is distinct from most East/Midwest US strains (P51) and most post-2000 strains (Nsp2, Nsp3, and Ssa3), which may be a contributing factor in PHF vaccine failure [24,55].

There are outlier strains which do not fit the geographical and temporal patterns. These include 081 [20,22], the Kentucky strain OV [22], and the Kentucky strain Herodia. Unique sequences in other *N. risticii *strains, such as TN02-1 (P51, Nsp2, and Nps3), KY03-3 (Nsp2), IL01-1 (Nsp3), and OH10-1 (Ssa3), suggest that variation contrary to the popular geographical and temporal influences may be more widespread. When additional contemporary sequences and sequences from more varied geographic regions become available, these analyses are expected to improve.

Possible explanations for extensive DNA sequence variation within *Neorickettsia *include the defective DNA repair systems in both *N. risticii *and *N. sennetsu *[37,38]. This would result in higher mutation rates for *Neorickettsia *[56], which would agree with the temporal changes and the production of outlier strains of *N. risticii*. P51 variation showed substantial geographical association, suggesting these variations were selected under local environmental pressures. It is possible that geographical association of *N. risticii *sequence variation is due to *N. risticii *strains being selected within essential reservoir trematode populations. In addition, diverse *N. risticii *strains may have emerged due to selective pressures inflicted on the infected trematodes and/or on the trematodes' hosts [4-9,57-59]. Humoral immunity would thus not play any direct role in creating genetic diversity within *N. risticii *populations. Since *Neorickettsia *spp. are known (*N. risticii *and *N. helminthoeca*) and suspected to be vertically transmitted within their trematode hosts [8,13,60], mammalian infection is not expected to be required for maintaining *Neorickettsia *in the natural environment.

Regardless the cause, this genetic variation would result in increased *N. risticii *survival as a species. *N. risticii *surface protein genetic diversity revealed in the present study will help in understanding variations in PHF virulence and clinical signs. It may also be possible to use this new molecular knowledge for vaccine development. It would, however necessitate taking into account that the pathogen is an obligate intracellular pathogen, indicating that not only humoral immune responses, but also cell-mediated immunity would play an active role in preventing bacterial infection [61-63].

Genes encoding the two original Ssas, called P85 (90-12) and P50 (25-D) are most related to *ssa1 *from *N. risticii *Illinois [24,31,38,55], but they also show similarities to *ssa2 *and the non-coding region between *ssa1 *and *ssa2 *using blastn. Although both are Maryland isolates, the 25-D strain was isolated six years earlier than the 90-12 strain [31], suggesting both temporal variation and the potential development of chimeras of multiple Ssas and non-coding regions in P50, P85, and post-2000 Ssa1 (due to the similarities of PA-1 and OH07-1 Ssa1 fragments to P85). It is possible that the high variability of Ssa1 may have prevented PA-1 Ssa1 from being identified by proteomics. However, there is the obvious lack of large numbers of peptides identified by proteomics for Ssas in *N. risticii *Illinois using the isogenic Illinois strain sequence data and in *N. sennetsu *using Miyayama isogenic strain data [39]. It is likely that Ssas are not a dominant surface protein in mammalian cells.

In conclusion, our data demonstrate the variety present within major surface proteins of *N. risticii*, and they suggest conservation among geographical regions and time periods. In addition, P51 is implicated as the major surface antigen of *N. risticii*. These data will be valuable in developing better diagnostic methods and may help in the development of more efficacious vaccines.

## Competing interests

The authors declare that they have no competing interests.

## Authors' contributions

KEG drafted the manuscript, designed primers, performed PCR and overall sequence analyses, and created secondary structures and dendrograms. GP designed primers, performed PCR, and performed preliminary sequence analyses. SM performed all SDS-PAGE and Western blotting experiments and gathered clinical data. YR edited the manuscript and supervised all research. All authors read and approved the final manuscript.

## Supplementary Material

Additional file 1 **Supplemental Table 1. Primers utilized for PCR amplification**. Word document demonstrating primers utilized for PCR amplification of ***p51***, ***nsp2***, ***nsp3***, ***ssa1***, and ***ssa3***. Click here for file
