# Supplementary material for: Neorickettsia risticii surface-exposed proteins: proteomics identification, recognition by naturally-infected horses, and strain variations
Source: Vet Res. 2011 Jun 2;42(1):71. doi: 10.1186/1297-9716-42-71 (PMC3127766; doi:10.1186/1297-9716-42-71)
Supplement: Additional file 1 — Supplemental Table 1. Primers utilized for PCR amplification. Word document demonstrating primers utilized for PCR amplification of p51, nsp2, nsp3, ssa1, and ssa3. [file 1297-9716-42-71-S1.DOC]

**Additional Table 1. Primers utilized for PCR amplification**.

| **Primer^a^** | **Direction** | **Sequence (5′-3′)** | **Primer pair(s)** | **Gene(s) amplified** |
| --- | --- | --- | --- | --- |
| GP38 | Forward | GGTTAGTTCTTTGCATTTTACTG | GP40 | *nsp2* |
| GP40 | Reverse | CAAAGTAGCGTGCAGTATATC | GP38 | *nsp2* |
| GP42 | Forward | TACCACGACTTCAGTGCTG | GP43 | *nsp2*, NRI_0840, *nsp3* |
| GP43 | Reverse | CTTCTTCAGCGAAACCTTC | GP42 | *nsp2*, NRI_0840, *nsp3* |
| GP41 | Forward | CTTGACGATGGACTTCTTG | GP39 | *nsp3* |
| GP39 | Reverse | CACAATTAGGACCGCAAC | GP41 | *nsp3* |
| Nsp1-743F | Forward | CACACAATATTGAAGCTGGTATAG | Nsp2-371R | *nsp1*, *nsp2* |
| Nsp2-371R | Reverse | CTTTCAGCGAGCTTACCTG | Nsp1-743F | *nsp2* |
| Nsp2-225F | Forward | CTATCTTAATGGTACTGTGATAAG | Nsp2/3-NCR-R | *nsp2*, NRI_0840 |
| Nsp2/3-NCR-R | Reverse | GTTCACCTCTTTGAAGTTTCATAG | Nsp2-225F | *nsp2*, NRI_0840 |
| Nsp2-231F-SF | Forward | CAATGGTACTGTGATAAGAGAATTC | Nsp2/3-NCR-R | *nsp2*, NRI_0840 |
| PER51-7 | Forward | tgtataaacttagcaagatattac | TM6 | *p51* |
| TM6 | Reverse | CAGCGATGGAAGATACATC | PER51-7 | *p51* |
| PER51-14 | Reverse | ACACTTGGTGTTAATGTAAGG | PER51-7 | *p51* |
| 51K-F7 | Forward | GTCTTCCAAAGATCGATGTCC | 51K-F7 | *p51* |
| 51K-R5 | Reverse | ttccgtaaccggtttcaaag | PER51-7 | *p51* |
| KM324 | Forward | CCGGCTGTTGAAAAAACGACATCA | KM325 | *p51* |
| KM325 | Reverse | AGCTCATACGTGCTTCCAGTGATG | KM324 | *p51* |
| KM017 | Forward | GTAACATTCGGAGAGAAGGGTTC | KM018 | *p51*, NRI_0234 |
| KM018 | Reverse | GAGAACAAGATTATAGGGATCCAAGT | KM017 | *p51*, NRI_0234 |
| 838-1a | Forward | GGTAAGGATGAAGCAAAAGCAGTAC | 838-4, 840-2 | *ssa1*, *ssa2*, *ssa3* |
| 838-4 | Reverse | CTGGTGCATAGTGCACTTCC | 838-1a | *ssa1* |
| 840-1 | Forward | CTAGTGCATCAAAAGGCGTGAG | 840-2 | *ssa3* |
| 840-2 | Reverse | CATTACCTGGACTTTCGAACAGC | 838-1a, 840-1 | *ssa1*, *ssa2*, *ssa3* |
| NCR839/840-1 | Forward | CATAACTTAGGGCTACTATCCC | 840-3, NCR840/841-1 | *ssa3* |
| 840-3 | Reverse | GTGAGAACATTGCCTACTTTATC | NCR839/840-1 | *ssa3* |
| 840-3F | Forward | GATAAAGTAGGCAATGTTCTCAC | NCR840/841-1 | *ssa3* |
| NCR840/841-1 | Reverse | CTTGTTATGGTAACCTGCTTG | NCR839/840-1, 840-3F | *ssa3* |

^a^Primers PER51-7, PER 51-14, 51K-F7, and 51K-R5 were designed by Dr Chunbin Zhang. Primers KM0324, KM0325, KM017, and KM018 were designed by Dr Koshiro Miura.
